# Peritruncal Coronary Endothelial Cells Contribute to Proximal Coronary Artery Stems and Their Aortic Orifices in the Mouse Heart

**DOI:** 10.1371/journal.pone.0080857

**Published:** 2013-11-21

**Authors:** Xueying Tian, Tianyuan Hu, Lingjuan He, Hui Zhang, Xiuzhen Huang, Robert E. Poelmann, Weibo Liu, Zhen Yang, Yan Yan, William T. Pu, Bin Zhou

**Affiliations:** 1 Key Laboratory of Nutrition and Metabolism, Institute for Nutritional Sciences, Shanghai Institutes for Biological Sciences, Graduate School of the Chinese Academy of Sciences, Chinese Academy of Sciences, Shanghai, China; 2 Department of Anatomy and Embryology, Leiden University Medical Center, Leiden, The Netherlands; 3 Ningbo 2nd Hospital, Ningbo, China; 4 Department of Cardiology, Zhongshan Hospital, Fu Dan University, Shanghai, China; 5 Department of Cardiology, Boston Children’s Hospital, Boston, Massachusetts, United States of America; Peking University, China

## Abstract

Avian embryo experiments proved an ingrowth model for the coronary artery connections with the aorta. However, whether a similar mechanism applies to the mammalian heart still remains unclear. Here we analyzed how the main coronary arteries and their orifices form during murine heart development. Apelin (*Apln*) is expressed in coronary vascular endothelial cells including peritruncal endothelial cells. By immunostaining, however, we did not find Apln expression in endothelial cells of the aorta during the period of coronary vessel development (E10.5 to E15.5). As a result of this unique expression difference, Apln^CreERT2/+^ genetically labels nascent coronary vessels forming on the heart, but not the aorta endothelium when pulse activated by tamoxifen injection at E10.5. This allowed us to define the temporal contribution of these distinct endothelial cell populations to formation of the murine coronary artery orifice. We found that the peritruncal endothelial cells were recruited to form the coronary artery orifices. These cells penetrate the wall of aorta and take up residence in the aortic sinus of valsalva. In conclusion, main coronary arteries and their orifices form through the recruitment and vascular remodeling of peritruncal endothelial cells in mammalian heart.

## Introduction

Coronary arteries connect to the aorta and supply oxygenated blood to the heart muscle [1-3]. For most of the last century, the coronary arteries were believed to grow out of the aorta, forming the coronary artery orifices and proximal coronary arteries by sprouting angiogenesis [4,5]. Experiments in avian embryos later questioned this dogma and suggested a reverse model in which coronary arteries forms from the preliminary coronary plexuses of the developing heart and grow into the aortic wall to form the coronary orifices and proximal coronary arteries [6-9]. In the key experiment, quail proepicardium was transplanted into the pericardial cavity of chicken embryos. Quail endothelial cells from transplantation were found to form the proximal part of the two main coronary arteries connecting aorta endothelium[8]. However, at present there is no data about whether a similar mechanism accounts for the coronary artery orifice formation in mammalian hearts. In large part, this has been due to the lack of proper genetic tools that distinguish coronary from aortic endothelia. 

 Here, we took advantage of a new inducible Cre genetic lineage tracing tool, Apln^CreERT2^, that allows us to distinguish endothelial cells of the coronary vasculature of the developing heart from those of the aortic wall. Our data demonstrate that the coronary artery stems and orifices are largely derived from the coronary vasculature of the developing heart, which grow into the aorta to communicate with the systemic blood supply.

## Methods

### Ethics Statement

This study was carried out in strict accordance with the guidelines in the Institutional Animal Care and Use Committee (IACUC) of the Institute for Nutritional Sciences, Shanghai Institutes for Biological Sciences, Chinese Academy of Sciences (Approved protocol number 2011-AN-2).

### Mice

Apln^CreERT2/+^, Apln^LacZ/+^, Wt1^CreERT2/+^, and Rosa26^RFP/+^ mice were described previously [10-13]. All mice were used in accordance with the guidelines of the Institutional Animal Care and Use Committee of the Institute for Nutritional Sciences, Shanghai Institutes for Biological Sciences, Chinese Academy of Sciences. Mice were maintained on a C129/C57BL6/J mixed background. Tamoxifen (Sigma) was dissolved in corn oil (0.1 - 0.15 mg/g). and introduced by gavage at the indicated time The date of the vaginal plug was designated as embryonic day 0.5 (E0.5). 

### Immunostaining

Immunostaining was conducted as previously described [14]. Briefly, embryonic and adult hearts were fixed in 4% PFA in PBS for 30 min and 2 h at 4°C, respectively. After washing in PBS, hearts were dehydrated in 30% sucrose at 4°C overnight. Specimens were embedded in OCT and quickly frozen in dry ice. Frozen tissues were sectioned at 10 μm thickness. Sections were blocked with 5% normal donkey serum in 0.2%Triton X-100 PBS for 30 min at room temperature. The specimens were incubated with rabbit anti-estrogen receptor (ESR, Abcam, ab27595), mouse anti-alpha smooth muscle actin (aSMA, Sigma, F3777), rabbit anti-RFP (Rockland, 600-401-379), rabbit anti-beta Gal (MP, 55976) and rat anti-PECAM antibodies (BD Pharmingen, 553370) in a humid chamber at 4°C overnight, and then washed three times in PBS for 5 min. Alexa Fluor secondary antibodies (Invitrogen) were added for 30 min at room temperature. HRP-conjugated or biotin-conjugated antibodies with tyramide signal amplification (PerkinElmer) were used to enhance signals of weakly staining antibodies. After a final rinse in PBS, the specimens were counterstained with 4’, 6-diamidino-2-phenylindole (DAPI) and coverslipped. Images were acquired using a Zeiss LSM510 confocal microscope, a Leica M165 FC stereo microscope, and a Olympus BX53 microscope.

### X-gal staining

X-gal staining was conducted as previously described with some modifications [15]. Embryos were dissected in PBS and fixed with 0.2% glutaraldehyde in PBS for 30 minutes. Then hearts or thoraxes of embryos were washed in X-gal washing buffer and stained in X-gal staining solution (1mg/ml X-gal) at 37°C over night. Subsequently, embryos were washed 3 times in washing buffer. Wholemount pictures were taken using a stereo microscope (Leica, M165 FC).

## Results

**Figure 1:**
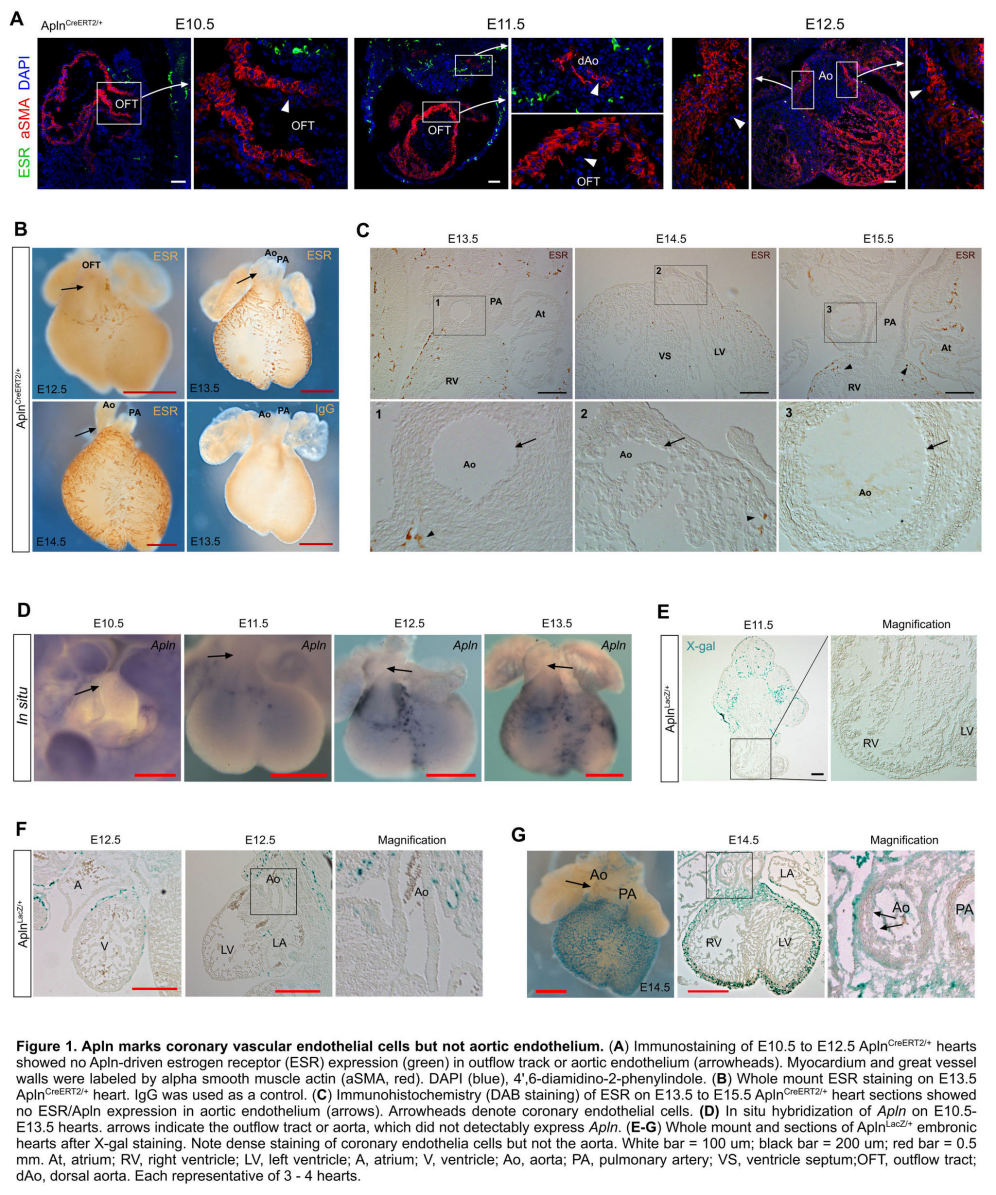
Apln marks coronary vascular endothelial cells but not aortic endothelium. (**A**) Immunostaining of E10.5 to E12.5 AplnCreERT2/+ hearts showed no Apln-driven estrogen receptor (ESR) expression (green) in outflow track or aortic endothelium (arrowheads). Myocardium and great vessel walls were labeled by alpha smooth muscle actin (aSMA, red). DAPI (blue), 4',6-diamidino-2-phenylindole. (**B**) Whole mount ESR staining on E13.5 AplnCreERT2/+ heart. IgG was used as a control. (**C**) Immunohistochemistry (DAB staining) of ESR on E13.5 to E15.5 AplnCreERT2/+ heart sections showed no ESR/Apln expression in aortic endothelium (arrows). Arrowheads denote coronary endothelial cells. (**D**) In situ hybridization of *Apln* on E10.5-E13.5 hearts. Arrows indicate the outflow tract or aorta, which did not detectably express *Apln*. (**E**-**G**) Whole mount and sections of AplnLacZ/+ embryonic hearts after X-gal staining. Note dense staining of coronary endothelia cells but not the aorta. Arrows indicate aorta endothelium that is negative for Apln expression. White bar = 100 um; black bar = 200 um; red bar = 0.5 mm. At, atrium; RV, right ventricle; LV, left ventricle; A, atrium; V, ventricle; Ao, aorta; PA, pulmonary artery; VS, ventricle septum;OFT, outflow tract; dAo, dorsal aorta. Each representative of 3 - 4 hearts.

Apln is specifically expressed in vascular endothelial cells of embryonic and adult hearts [10,16]. To examine Apln expression in embryos, we used Apln^CreERT2/+^ and Apln^LacZ/+^ knockin mouse lines [10,12], and stained for estrogen receptor (ESR) or beta-galactosidase as surrogates for Apln expression. ESR staining on embryonic sections and wholemount hearts showed Apln is specifically expressed in vascular endothelial cells at examined stages (E10.5 to E15.5, Figure 1A-1C), which is in consistence with previous work [10,12,16]. ESR staining of Apln^CreERT2/+^ embryonic hearts showed that Apln is not expressed in endothelia of aorta through all examined stages of coronary development (E10.5 to E15.5, Figure 1A-C). To confirm the immunostaining result, we used in situ hybridization on wild type hearts from E10.5 to E13.5 and found Apln is not expressed in endothelium of outflow tract or aorta at these stages (Figure 1D). These results were independently reinforced by wholemount X-gal staining of Apln^LacZ/+^ embryonic hearts, which did not detect Apln-driven LacZ expression in aortic endothelium (Figure 1E-1G). 

**Figure 2:**
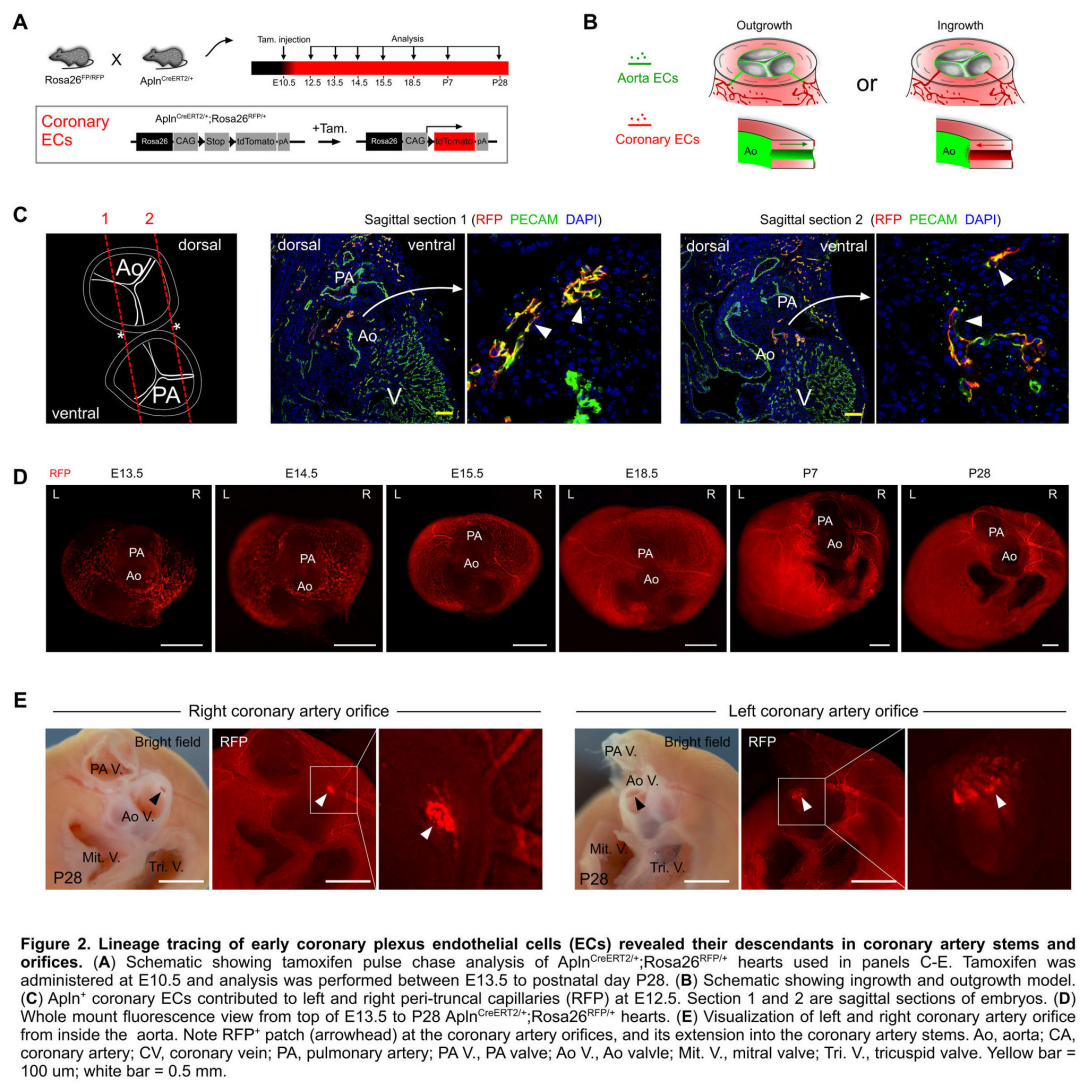
Lineage tracing of early coronary plexus endothelial cells (ECs) revealed their descendants in coronary artery stems and orifices. (**A**) Schematic showing tamoxifen pulse chase analysis of AplnCreERT2/+;Rosa26RFP/+ hearts used in panels C-E. Tamoxifen was administered at E10.5 and analysis was performed between E13.5 to postnatal day P28. (**B**) Schematic showing ingrowth and outgrowth model. (**C**) Apln+ coronary ECs contributed to left and right peri-truncal capillaries (RFP) at E12.5. Section 1 and 2 are sagittal sections of embryos. (**D**) Whole mount fluorescence view from top of E13.5 to P28 AplnCreERT2/+;Rosa26RFP/+ hearts. (**E**) Visualization of left and right coronary artery orifice from inside the aorta. Note RFP+ patch (arrowhead) at the coronary artery orifices, and its extension into the coronary artery stems. Ao, aorta; CA, coronary artery; CV, coronary vein; PA, pulmonary artery; PA V., PA valve; Ao V., Ao valvle; Mit. V., mitral valve; Tri. V., tricuspid valve. Yellow bar = 100 um; white bar = 0.5 mm.

**Figure 3:**
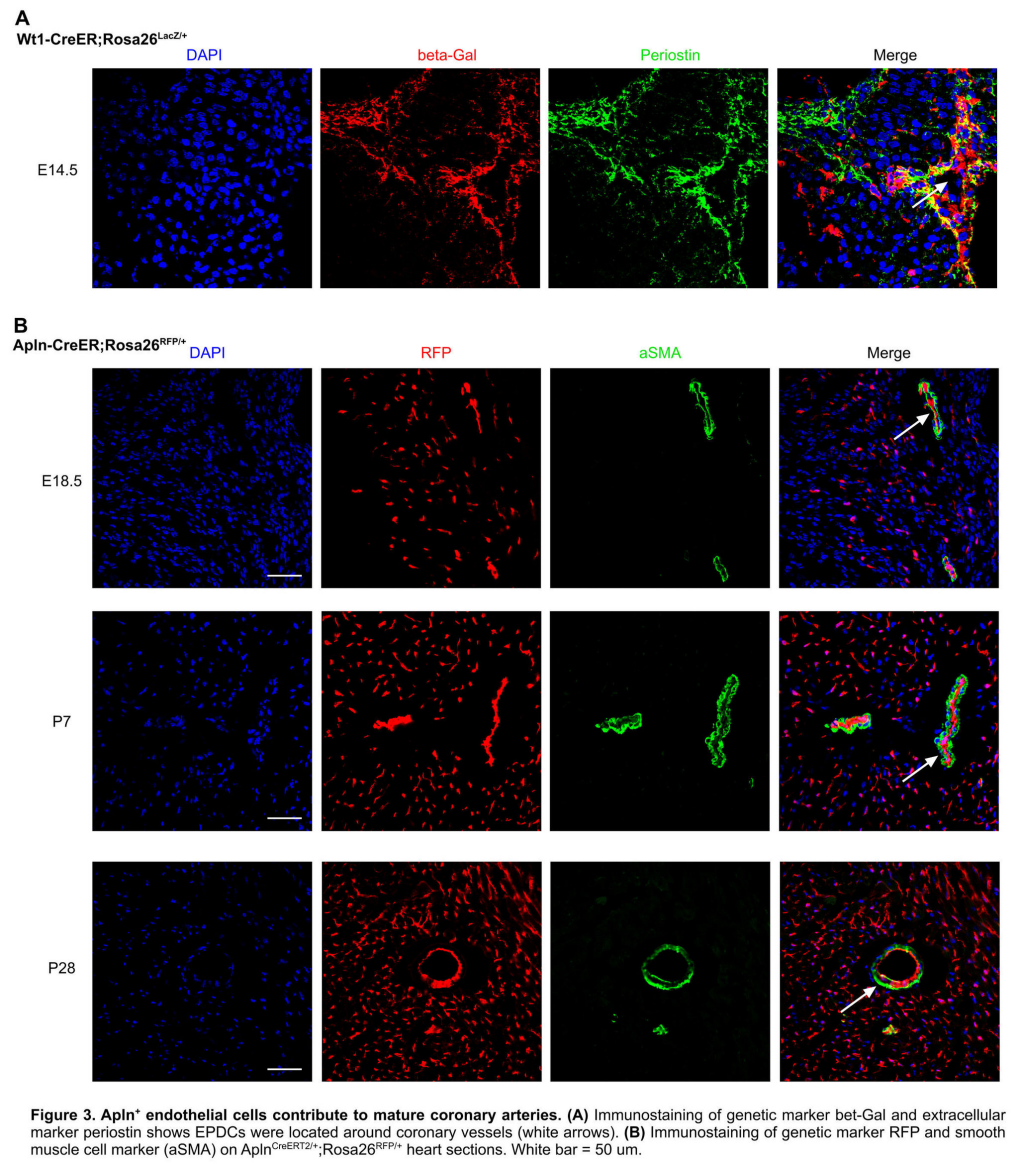
Apln+ endothelial cells contribute to mature coronary arteries. (**A**) Immunostaining of genetic marker bet-Gal and extracellular marker periostin shows EPDCs were located around coronary vessels (white arrows). (**B**) Immunostaining of genetic marker RFP and smooth muscle cell marker (aSMA) on AplnCreERT2/+;Rosa26RFP/+ heart sections. White bar = 50 um.

**Figure 4:**
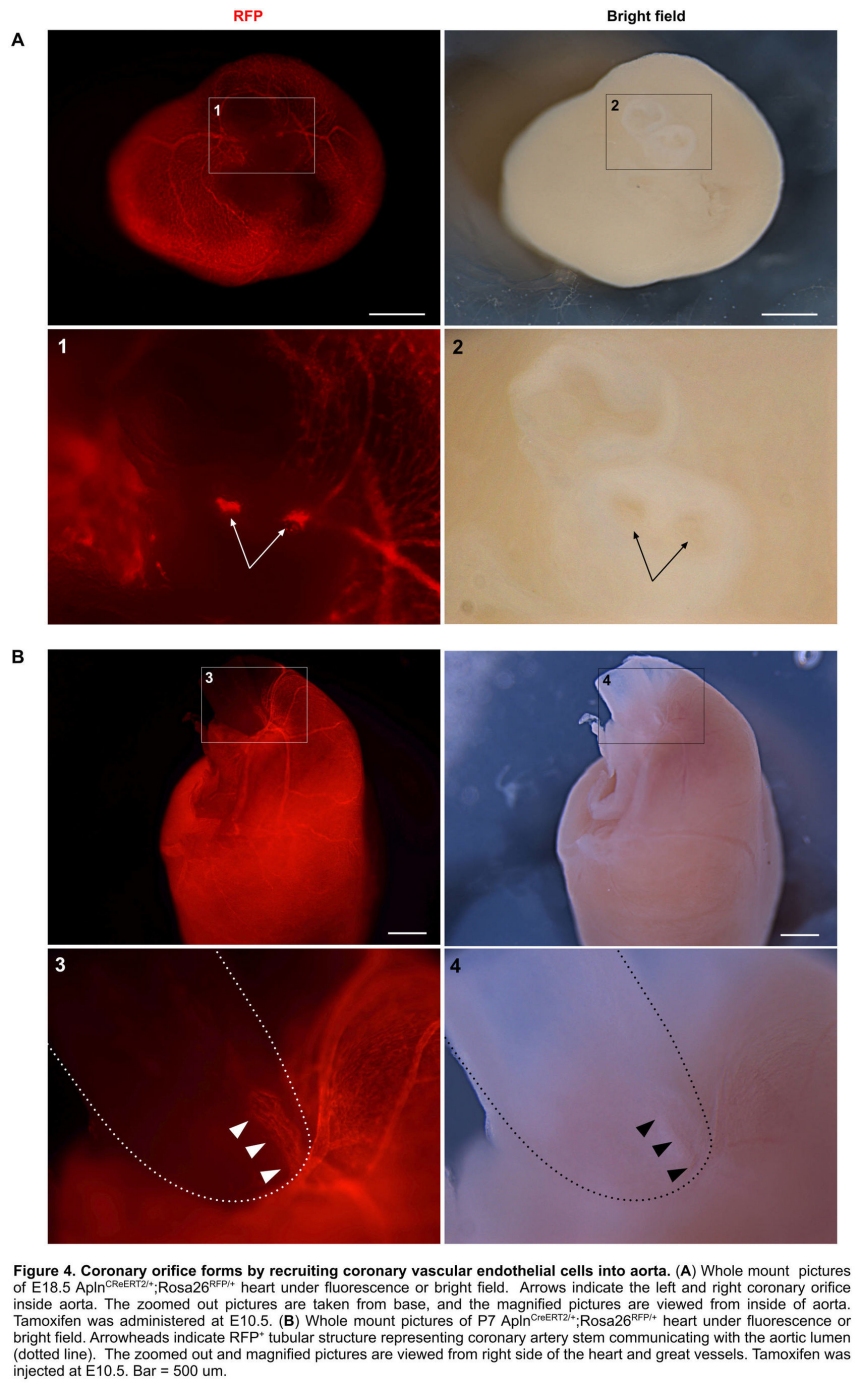
Coronary orifice forms by recruiting coronary vascular endothelial cells into aorta. (**A**) Whole mount pictures of E18.5 AplnCreERT2/+;Rosa26RFP/+ heart under fluorescence or bright field. Arrows indicate the left and right coronary orifice inside aorta. The zoomed out pictures are taken from base, and the magnified pictures are viewed from inside of aorta. Tamoxifen was administered at E10.5. (**B**) Whole mount pictures of P7 AplnCreERT2/+;Rosa26RFP/+ heart under fluorescence or bright field. Arrowheads indicate RFP+ tubular structure representing coronary artery stem communicating with the aortic lumen (dotted line). The zoomed out and magnified pictures are viewed from right side of the heart and great vessels. Tamoxifen was injected at E10.5. Bar = 500 um.

**Figure 5:**
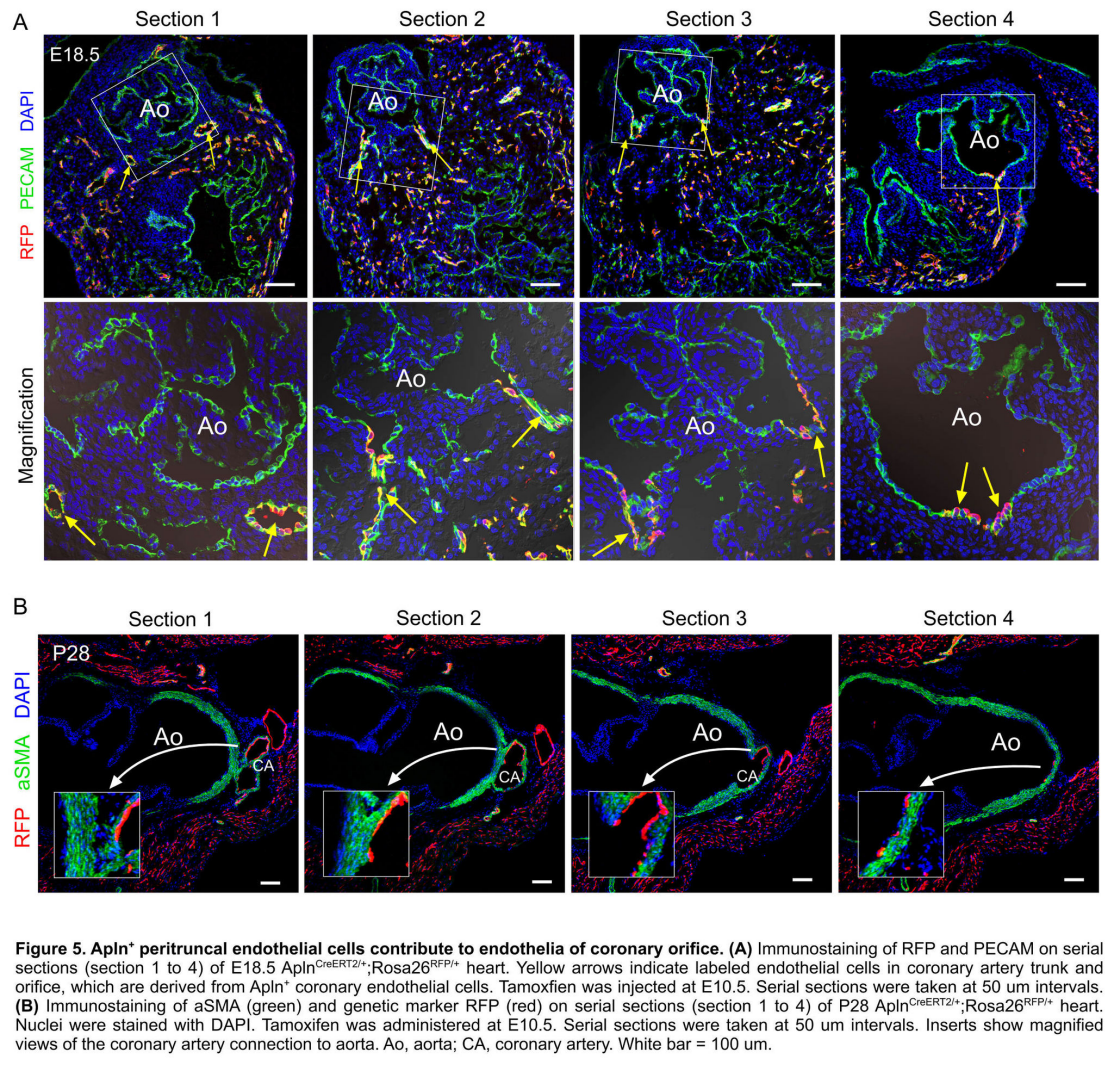
Apln+ peritruncal endothelial cells contribute to endothelia of coronary orifice. (**A**) Immunostaining of RFP and PECAM on serial sections (section 1 to 4) of E18.5 AplnCreERT2/+;Rosa26RFP/+ heart. Yellow arrows indicate labeled endothelial cells in coronary artery trunk and orifice, which are derived from Apln+ coronary endothelial cells. Tamoxfien was injected at E10.5. Serial sections were taken at 50 um intervals. (**B**) Immunostaining of aSMA (green) and genetic marker RFP (red) on serial sections (section 1 to 4) of P28 AplnCreERT2/+;Rosa26RFP/+ heart. Nuclei were stained with DAPI. Tamoxifen was administered at E10.5. Serial sections were taken at 50 um intervals. Inserts show magnified views of the coronary artery connection to aorta. Ao, aorta; CA, coronary artery. White bar = 100 um.

 Positive Apln expression in the developing coronary plexus over the heart and negative Apln expression in aortic endothelium indicated that Apln^CreERT2/+^ lineage tracing would be informative on the contribution of the early coronary plexus of the developing heart to the coronary artery stems and orifices (Figure 2A). Based on models proposed by previous avian work [4-8], we evaluated two models for coronary stem and orifice formation in mouse developing heart: an outgrowth model in which the unlabeled aortic endothelium spreads out to form the coronary artery stems and orifices through angiogenesis; and an ingrowth model in which AplnCreERT2-labeled peritruncal coronary endothelial cells are first recruited into the aorta wall and then coalesce and remodel to form the proximal coronary arteries and their orifices in the sinuses of Valsalva of the aorta (Figure 2B). For all studies, we induced Cre-mediated recombination by E10.5 tamoxifen injection, which specifically label coronary vascular plexus by genetic marker RFP at early stages (before E12.5) [12]. By immunostaining of the genetic lineage tracing marker (RFP) and endothelial cell marker (PECAM) on E12.5 Apln^CreERT2/+^;Rosa26^RFP/+^ heart sections, we detected the genetically labeled peritruncal capillary plexus around the aorta, especially in the region close to the right and left facing aortic sinuses [17] (Figure 2C). From E13.5 to postnatal stages, the main coronary arteries gradually emerge, and they become readily detectable after E14.5 (Figure 2D). By E10.5 pulse tamoxifen induction, almost all endothelium of the main coronary arteries and their branches were genetically labeled, as detected at later stages (Figure 2D), suggesting that endothelial cells were first recruited to the peritruncal region where they contributed to form the proximal coronary arteries. The coronary arteries marked by Apln^+^ coronary vascular plexus mature after E14.5 through vascular modeling and recruitment of vascular smooth muscle cells from epicardium derived cells, EPDCs labeled by Wt1-CreER (Figure 3). On the inner lining of the aorta, we detected labeled descendants of peritruncal coronary endothelial cells (RFP^+^) at E15.5, E18.5, postnatal day 7 (P7), P28 stages (Figure 2E and Figure 4 and data not shown). The labeled coronary endothelial cells spanned the thick aortic wall, constituting virtually the entire coronary orifice (Figure 4). To provide further evidence at single cell resolution, we sectioned these Apln^CreERT2/+^;Rosa26^RFP/+^ hearts for immunostaining of genetic and vascular markers. By studying serial sections, we verified that the endothelium lining the orifices in the aorta was derived from early Apln^+^ coronary vascular plexus (Figure 5). 

## Discussion

 Previous work based on avian models suggested that the endothelium of the coronary artery originates from (pro)epicardium [18,19] and sinus Venosus [8], and eventually grows into the aorta, thereby forming the coronary arterial orifices [6,7,20]. However, studies of coronary artery formation in mammals do not often lead to same conclusions, so that it is important to revisit this question in mammals. Our work is consistent with the coronary artery ingrowth model, and extends this model by showing that the peritruncal endothelial cells constitute virtually all coronary artery stems and two coronary orifices in the aorta. Lineage tracing data also suggested that coronary artery stems initially form by recruitment of peritruncal endothelial cells, subsequently followed by a remodeling and maturation process with addition of smooth muscle cells.

 The peritruncal coronary plexus forms at E12.5, providing multiple vessels that could potentially connect to the aorta. In both chicken and mouse models, peritruncal vascular endothelial cells penetrated the facing left and right sinuses. Only these two coronary artery strands penetrating the aortic wall finally develop into the coronary artery stems and orifices. In some chicken embryos, several endothelial strands penetrated the posterior (noncoronary) sinus, and they eventually disappear [8,9]. The regional signaling that governs the establishment, penetration, maintenance, and growth of these two coronary artery strands remains largely unknown. It was indicated by chicken model that proper orifice development is associated with penetration of epicardium-derived cells (EPDCs) that produce Fas ligand as an apoptotic inductor at sites of coronary ingrowth [20]. Since disruption of EPDCs contribution show abnormalities in coronary ingrowth and orifice formation [20] and also EPDCs exhibit pro-angiogenic properties [14], it would be important to investigate the secreting profile of penetrating EPDCs for peritruncal vascular angiogenesis. Previous work showed that EPDCs contribute to most smooth muscle cell fate [13,21,22], we reasoned that their in situ trans-differentiation into smooth muscle could be responsible for the consolidation of the main arteries. Alternatively, possible cues for guidance of ingrowth and subsequent persistence of the proximal coronary artery stems could be the parasympathic innervation by neural crest cells, as ablation of neural crest cells causes abnormalities in coronary orifice formation [23].

 Our study provided a useful tool for genetically marking the coronary plexus and subsequent coronary artery stems and orifices, allowing visualization of the entire process of coronary artery orifice development under normal and pathological conditions such as anomalous origin of the right or left coronary artery (AORCA or AOLCA) [24,25]. The ability to monitor this process will facilitate future mechanistic studies of the signals that govern it. In addition, differential labeling of nascent versus established vasculature by Apln^CreERT2^ will likely make it useful to study vascular growth patterns in other organs or tissues under normal and pathophysiological conditions such as ischemic disease, vascular regeneration and tumor angiogenesis.
